# Climatic niche variation in genetically distinct populations throughout the annual cycle for a migratory parulid bird, *Cardellina pusilla*

**DOI:** 10.7717/peerj.21540

**Published:** 2026-08-17

**Authors:** Genaro Rodríguez Otero, Christen Bossu, Marius Somveille, Sergio Gómez-Villaverde, Kristen Ruegg, Blanca E. Hernández-Baños

**Affiliations:** 1 Posgrado en Ciencias Biológicas, Universidad Nacional Autónoma de México, Mexico City, Mexico; 2 Department of Biology, Colorado State University, Colorado State University, Fort Collins, CO, United States of America; 3 School of Environmental Sciences, University of East Anglia, Norwich, United Kingdom; 4 Observatorio de Aves de Tlaxiaco (OATL), Oaxaca, Mexico; 5 Museo de Zoología, Departamento de Biología Evolutiva, Facultad de Ciencias, Universidad Nacional Autónoma de México, Mexico City, Mexico

**Keywords:** Climatic, Niche, Variation, Annual cycle, *Cardellina pusilla*, Migratory

## Abstract

At the intraspecific level, environmental heterogeneity can create opportunities for local adaptation and population differentiation. However, studying the effects of environmental heterogeneity in migratory animals is challenging due to the complexity of their natural history, which is conditioned by their long journeys each year. *Cardellina pusilla* is a widespread Neotropical migratory bird that comprises six genetically distinct populations. We conducted a detailed study of the potential for differentiation among those populations in their climatic niche space and preferences across the annual cycle. Previous studies had focused mainly on the breeding part of their range, leaving the non-breeding range under-sampled. We filled in those sampling gaps with genetic samples in key regions of the non-breeding grounds to analyze variation in the realized climatic niche among the six genetically distinct populations. We found that niche breadth was wider during the non-breeding season than the breeding season. There were also clear differences in breeding niches among populations, with that of the Eastern Boreal population being the most differentiated from the other populations. The non-breeding niches were more similar among populations, but the Eastern Boreal population still showed low overlap with the other populations. Comparisons from seasonal climatic niche overlap and potential migratory destinations revealed that several populations (*e.g.,* Coastal California, Pacific Northwest) would experience a better climatic match if they occupied non-breeding grounds used by other populations, yet they do not do so. This pattern is consistent with recent theoretical frameworks suggesting that philopatry, site fidelity and energy efficiency are stronger drivers of migratory connectivity than climatic niche. Our findings demonstrate substantial intraspecific niche variation across the annual cycle, that populations follow distinct migratory strategies, and that non-breeding ground fidelity may limit migratory animals’ ability to adjust to climate change. Conservation efforts should prioritize the protection of historically used non-breeding areas and recognize that population-specific vulnerabilities are shaped by migratory fidelity, not by potential climatic optima.

## Introduction

Environmental heterogeneity creates opportunities for local adaptation and divergence between populations, playing a crucial role in maintaining and generating biological diversity (Fitzpatrick & Keller, 2015; Savolainen, Lascoux & Merilä, 2013). This process begins when populations are exposed to a heterogeneous mosaic of environmental conditions, facilitating adaptation to local biotic and abiotic factors. Such local adaptation results in the differentiation of phenotypes and genotypes, which have profound ecological consequences. These traits often lead to non-random dispersal (Edelaar & Bolnick, 2012), influence dispersal behavior, and can establish or maintain geographic range boundaries (Kawecki & Ebert, 2004; Nakazawa, 2013; Nosil, Vines & Funk, 2005; Savolainen, Lascoux & Merilä, 2013; Wang, 2013). Given that local adaptation shapes dispersal and range limits, a detailed understanding of this process becomes essential for predicting how populations will respond to global environmental change (Greenberg, 2016; Janzen, 1967).

It has been difficult to study the effects of environmental heterogeneity on intraspecific differentiation in migratory animals because their natural history is shaped by conditions on their breeding grounds, their non-breeding grounds, and their movements between them (Chen et al., 2022; Helbig, 2003; Somveille et al., 2024; Winger et al., 2019). Migratory birds cope with extreme environmental changes at northern latitudes by traveling southwards, sometimes thousands of kilometers, to spend the non-breeding season in regions where the climate is milder and resources are more abundant (Alerstam & Bäckman, 2018; Helbig, 2003; Somveille et al., 2018; Winger et al., 2019). Some species follow similar climatic conditions across the annual cycle; these species are known as niche trackers or followers. Conversely, other species known as niche switchers migrate between seasonal grounds with different climatic conditions (Gómez et al., 2016; Joseph & Stockwell, 2000; Nakazawa et al., 2004; Martínez-Meyer, Peterson & Navarro-Sigüenza, 2004; Zurell et al., 2018). These two emerging migration behaviors are related to the evolutionary history, ecology and population dynamics of each species and often have been previously examined at the species level (Gómez et al., 2016; Gutiérrez Illán et al., 2022; Nakazawa et al., 2004; Martínez-Meyer, Peterson & Navarro-Sigüenza, 2004; Ochoa-González et al., 2023). However, it is possible that varied evolutionary and ecological processes could also occur among populations that studies at the species level might miss (Alvarado et al., 2022; Mota-Vargas & Rojas-Soto, 2016; Somveille et al., 2024; Zurell et al., 2018).

While understanding variation in environmental preferences in both time and space at the infraspecific level has important implications for conservation of migratory animals in the face of global change (Chen et al., 2022; Mota-Vargas & Rojas-Soto, 2016; Smith et al., 2019; Somveille et al., 2024), identifying the links between breeding and non-breeding grounds can be challenging. Researchers have gone to extraordinary lengths to develop methods to track animals throughout their impressive journeys and generate more precise data (Ruegg et al., 2014; Ruegg et al., 2021; Turbek, Scordato & Safran, 2018). At the same time, genomic data has allowed researchers to investigate how genetically distinct populations are distributed in space. For migratory species, this includes the degree of migratory connectivity (spatiotemporal linkages of populations between seasons; Webster et al., 2002), habitat preferences throughout the annual cycle, demographic trends, and population divergence (Pegan et al., 2025; Ruegg et al., 2014; Somveille et al., 2024; Turbek, Scordato & Safran, 2018). Together, migratory tracking, genetic, demographic and ecological data can help provide a more complete picture of population differentiation and contribute to more robust conservation strategies (Kirby et al., 2008; Runge et al., 2014).

Combining genomic data with climatic niche models can shed light on the potential for intraspecific variation in climate preferences across the entire annual cycle (Alvarado et al., 2022; Laube, Graham & Böhning-Gaese, 2015; Ponti et al., 2020; Zurell et al., 2018). Some research suggests that species niches evolve slowly, and as a result ecological differentiation between populations should be rare (Wiens, 2004). However, empirical evidence of intraspecific niche differentiation is growing, and there are theoretical studies that argue that the fundamental niche could differentiate faster when two populations are split by a barrier and adapt to new environmental conditions (Janzen, 1967; Smith et al., 2019). While both environmental and genetic drivers of alternative migratory phenotypes should eventually lead to genetic differentiation, the role of these processes in intraspecific divergence remains poorly understood (Turbek, Scordato & Safran, 2018).

Wilson’s Warbler, *Cardellina pusilla*, is a migratory warbler species whose genetic variation has been well studied (Ammon & Gilbert, 2020; Clegg et al., 2003; Irwin, Irwin & Smith, 2011; Kimura, Clegg & Lovette, 2002; Ruegg et al., 2014). In the most recent study, Ruegg et al. (2014) analyzed genomic data and identified six genetically distinct populations across the breeding range of the species: Coastal California, Pacific Northwest, California Sierra, Western Boreal, Basin Rockies and Eastern Boreal. The breeding grounds of the six populations are geographically separate, but they mix during the non-breeding season. A previous investigation addressed the differences in seasonal climatic niche between western and eastern groups of the *Cardellina pusilla* complex (Ruiz-Sánchez et al., 2015) and found partial climate differentiation between the groups in the breeding and non-breeding seasons; the western group (subspecies *pileolata* and *chryseola*) have a broader niche and climatic tolerance than the eastern group (subspecies *pusilla*). These results support the idea that the *Cardellina pusilla* complex is composed of highly divergent groups—an eastern form and a western form (Irwin, Irwin & Smith, 2011; Ruegg et al., 2014; Ruiz-Sánchez et al., 2015). In the current study we investigate in greater detail the potential for among-population differentiation in climate use and preferences through the annual cycle. Our aims were to: (i) analyze variation in the realized climatic niche among the six genetically distinct populations of *Cardellina pusilla*, and (ii) determine whether the populations within the species exhibit niche-following or niche-switching migratory behavior, in order to understand how this could shape the migratory connectivity and population trends of each population.

## Materials and methods

### Sample collection

We used the data set created by Ruegg et al. (2014), which generated the genoscape of the species, that is, a map of genetic variation across the geographic range of *Cardellina pusilla*. The data set and genoscape were created with feather and blood samples from 1,648 individuals from 68 locations across the breeding, non-breeding and migratory range of the species. To fill in some sampling gaps and increase the sample size from *C. pusilla*’s non-breeding range from Mexico to Panama, we incorporated 29 additional feather and blood samples provided by MoSI (Monitoreo de Sobrevivencia Invernal) stations in Sinaloa and Baja California Sur, Mexico that were collected between December 2020 and January 2021 (collection permit: Instituto Nacional de Ecología, SEMARNAT, México FAUT-0169). We also received feather samples from MoSI stations in other parts of Mexico (*n*= 14) and Honduras (*n*= 28). In addition, we included tissue samples (*n*= 14) and samples retrieved from the toe pad of the hallux (*n*= 27) of museum specimens collected across Mexico in the non-breeding season and vouchered in the Ornithology Collection from the Museo de Zoología “Alfonso L. Herrera” de la Facultad de Ciencias (MZFC), Universidad Nacional Autónoma de México (UNAM).

### DNA extraction, SNP genotyping and population assignment

For newly acquired samples from museum specimens and banding stations in the non-breeding grounds, we extracted genetic material from the tip of the calamus of one of the outer rectrices, blood collected *via* brachial venipuncture and preserved in Queen’s lysis buffer (Seutin, White & Boag, 1991), or tissues (muscle, heart or liver) preserved at −70 °C. These samples were genotyped using SNPtype Assays (Fluidigm Inc.) on a FluidigmTM 96.96 IFC controller using a panel of 96 loci that Ruegg et al. (2014) determined to be strongly diagnostic of distinct genetic units. We then used the EP1 Array Reader and Fluidigm’s automated Genotyping Analysis Software (Fluidigm Inc.) to call alleles with a confidence threshold of 90%. Each genotype was also visually inspected for potential irregularities and manually called, and uncertain genotype calls were removed from the analysis. Samples with missing genotypes in >10% of SNP assays were removed from the analyses. Each non-breeding individual sampled was assigned to the genetically distinct populations recovered by Ruegg et al. (2014) using the R package *rubias* (Moran & Anderson, 2018), as previously described in Rueda-Hernández et al. (2023). Specifically, *rubias* is a Bayesian hierarchical genetic identification method that incorporates population structure and accounts for variations in the number of genetically differentiated populations. The self-assessment function in *rubias* evaluates assignment accuracy by measuring the proportion of individuals from known genetically distinct breeding groups that are correctly assigned back to their group of origin. We defined a robust assignment as one with a posterior probability >0.8 of belonging to the inferred collection. Assignments with a posterior probability <0.8 were considered uncertain, and those individuals were excluded from subsequent analysis (Moran & Anderson, 2018). The six genetically distinct populations were Coastal California, California Sierra, Pacific Northwest, Western Boreal, Basin Rockies and Eastern Boreal, following Ruegg et al. (2020).

### Climatic seasonal niche modeling

Once we had assigned the population for the additional non-breeding birds sampled, we combined them with the samples from Ruegg et al. (2014). We then modeling their realized climatic niche in order to explore the relationships between the environmental conditions on the breeding and non-breeding grounds of the six genetically distinct populations (hereafter, we use this term interchangeably with the terms “genetic group” or “genetic population”). Taking advantage of genomic data and ecological niche modeling to analyze the relationships and patterns of migratory behaviour and connectivity at the population level, especially when the populations intermix, is a novel and interesting way to understand the forces that shape those processes and the natural history of migratory animals and very relevant to this study (De Saix et al., 2019; Ruegg et al., 2014; Saracco & Rubenstein, 2020). We used two types of data: the data set from genotyped individuals with certain assignment (banding stations and museum specimens described above) and data from eBird records that we downloaded from GBIF (GBIF.org, 2022). We took into account the full annual cycle to better characterize the realized climatic niche (Eyres, Böhning-Gaese & Fritz, 2017; Ponti et al., 2020). To do this, we first split the data set of genotyped individuals (393 individuals sampled from 33 locations) by date into those from the summer breeding season (June and July) and those from the winter non-breeding season (345 individuals sampled; November to February). We used these strict date ranges, which were based on abundance maps from eBird (Fink et al., 2022), to avoid including individuals that could still be migrating.

We then supplemented the genetic dataset with the eBird records from June–July (1,322 occurrences for the entire species). Since Ruegg et al. (2014) found that each genetically distinct population occupies geographically distinct areas on the breeding grounds, but overlap on the non-breeding grounds, an individual’s location is only a reliable indicator of its genetic group during the breeding season, and not during the non-breeding season. Thus, we only used eBird records from June–July that were within the established breeding range of each genetic group. The eBird records were carefully curated; we filtered to remove records with erroneous species identification, locations, dates, or with no available data. We also removed records from areas that the genetic analysis in Ruegg et al. (2014) identified as contact zones between two genetic populations’ breeding ranges. Although this does pose some risk of incompletely characterizing the realized climatic niche, it prevents the inclusion of individuals from an adjacent population due to the difficulty of assigning their genetic identity.

We performed niche calculations for the breeding and non-breeding areas separately, using the complete data set for the species as a whole and then splitting among the six genetic groups. We used WorldClim historic climate data from the monthly time series of precipitation and mean temperature, averaged from 1970–2000 with a spatial resolution of 2.5 arc minutes (Fick & Hijmans, 2017). For the breeding season, we averaged the climate data for June and July, and for the non-breeding season, we averaged the climate data for November, December, January and February. We only used temperature and precipitation to approximate the realized climatic niche because these variables have been shown to have the strongest influence on bird species distribution globally, and are the most restricting for species distributions at the continental scale (Araújo, Thuiller & Yoccoz, 2009; Ruegg et al., 2017). All climate data were managed using the *sp* (Bivand, Pebesma & Gomez-Rubio, 2013), *rgdal* (Bivand, Keitt & Rowlingson, 2022), *raster* (Hijmans, 2020), and *terra* (Hijmans, 2022) packages in R version 4.2.3 (R Core Team, 2023). Climate data were extracted for each individual at its exact geographical location then normalized using z-scores across the complete distribution area of *C. pusilla*. The realized climatic niche was then estimated for each season and each genetic population by projecting the occurrences into a two-dimensional climate space defined by temperature and precipitation to obtain a cloud of points (Ruegg et al., 2021). Following the modeling framework of Broennimann et al. (2012), we estimated niche density by creating a 2D kernel density of 50  × 50 pixels with the “kde2d” function (bandwidth of 1, keeping the top 95% of the kernel density and setting the rest to 0) of the *MASS R* package (Venables & Ripley, 2002) overlaid on the two-dimensional climate space. We calculated realized niche size (as a proxy for the niche breadth of each genetic group) as the number of pixels with a density above 0 across climate space for each season (Ruegg et al., 2021).

We used the *ecospat* package in R (Di Cola et al., 2017) to evaluate niche overlap between seasons for each population and between populations during a given season. We calculated the Schoener’s D and Hellinger’s I metrics which range from 0 (no niche overlap) to 1 (niches overlap completely; Warren, Glor & Turelli, 2008; Broennimann et al., 2012). For the niche overlap calculation, we corrected the occurrence densities of each population by the prevalence of the environments in their range (Broennimann et al., 2012). We present the results for the D metric in the main text and the results for the I metric in the Supplementary Materials, and we followed the categories of Rödder & Engler (2011) to interpret the overlap measures derived from the D metric (0–0.2 = no overlap or very low overlap; 0.2–0.4 = low overlap; 0.4–0.6 = moderate overlap; 0.6–0.8 = high overlap; 0.8–1 very high overlap). We also performed niche similarity tests (Warren, Glor & Turelli, 2008) to evaluate the statistical significance of the measured niche overlaps between seasons against null model climate niches.

To assess the measured differences or similarities between the climatic niches and evaluate whether a population is a niche tracker or niche switcher, we shuffled the non-breeding ground location among all groups and calculated the D and I metrics for every possible pairing, then compared those values against the observed seasonal niche overlap of the genetic population. For this, we created a 6 × 6 matrix with each population’s realized breeding niche as rows and those for the non-breeding season as columns, to obtain the niche overlap for all possible shifts in destination.

## Results

### New samples

**Figure 1:**
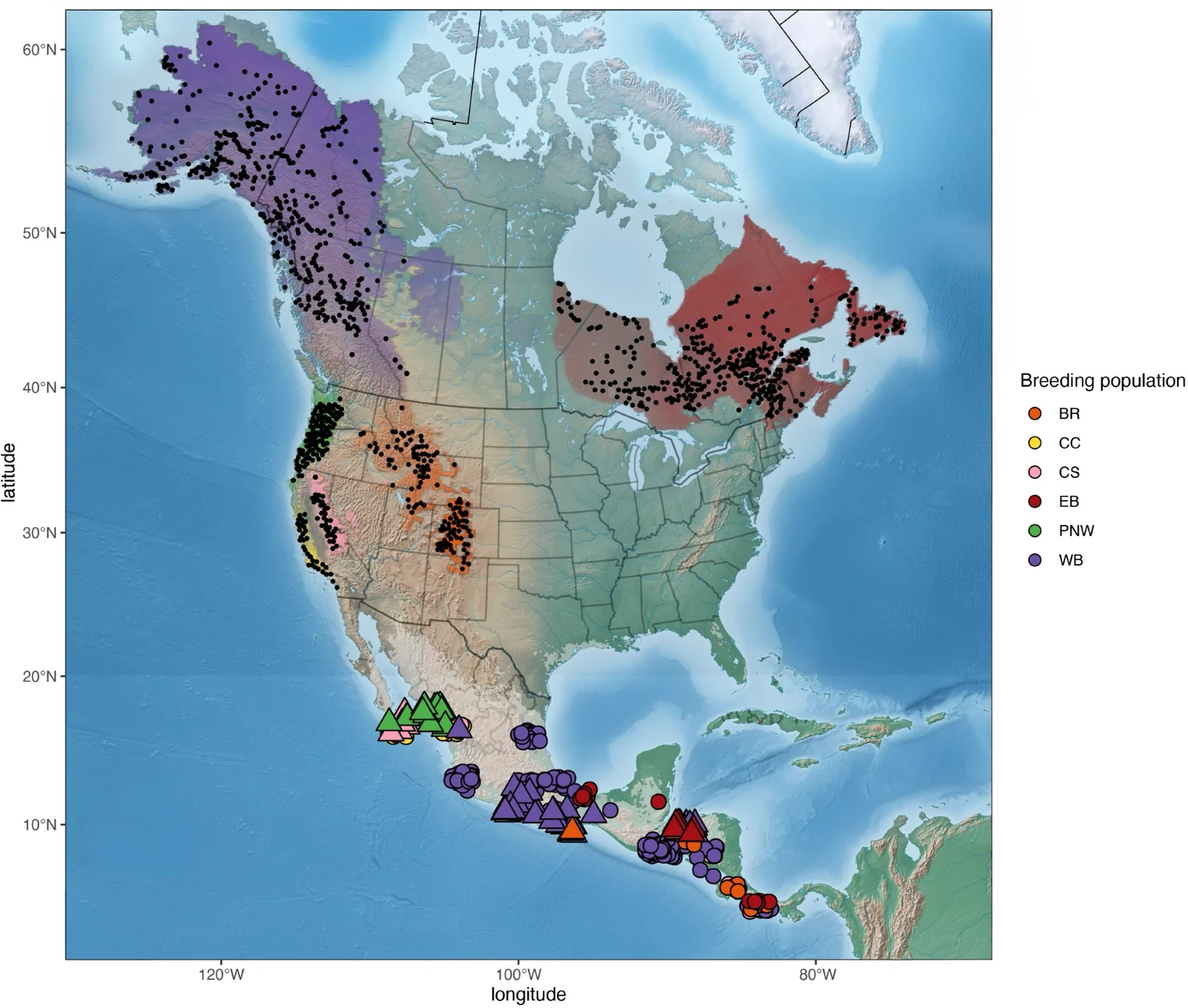
*Cardellina pusilla*’s genoscape with sampling localities in the breeding and nonbreeding grounds (based on Ruegg et al., 2014). In the breeding grounds we used genetic samples and eBird records from June to and July. In the nonbreeding grounds we only used genetic samples due to the fact that the populations mix in during the winter season, so eBird records cannot be used to determine genetic identity. The breeding populations to which the individuals belong are: BR (orange) = Basin Rockies, CC (yellow) = Coastal California, CS (pink) = California Sierra, EB (red) = Eastern Boreal, PNW (green) = Pacific Northwest, and WB (purple) = Western Boreal. Triangles represent the newly genotyped samples, while circles are represent samples genotype d by Ruegg et al. (2014).

Of the 107 newly genotyped non-breeding samples, 86 were assigned with a posterior probability >0.8 to one of the six genetically distinct populations within the species (Ruegg et al., 2014). Of the 86 samples that were assigned to a genetic group, five were assigned to the Eastern Boreal group, 47 to Western Boreal, three to Basin Rockies, 10 to California Sierra, 21 to Pacific Northwest, and no samples were assigned to Coastal California (Fig. 1).

### Realized climatic niche of the species and its genetically distinct populations

**Figure 2:**
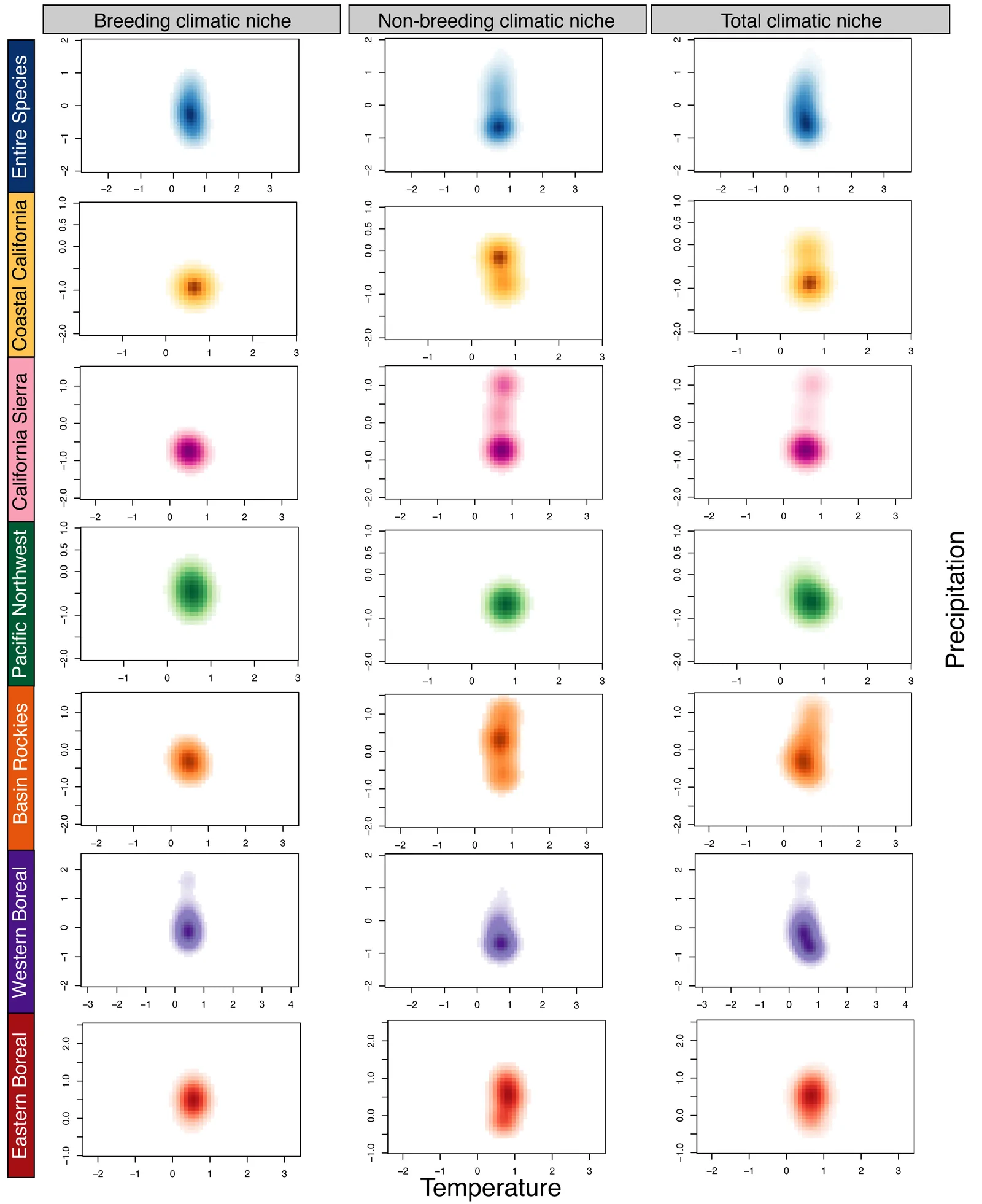
Realized climatic niches for the entire species and for each population in their breeding grounds, nonbreeding grounds and the seasonal niche overlap (breeding plus nonbreeding niches).

*Cardellina pusilla* occurs in strongly contrasting environments with extreme values of both temperature and precipitation throughout its annual cycle. Based on the climatic layers used, during the breeding season the temperature can get as low as −5 °C in some parts of its range and as high as 27 °C in other parts, with a mean of 13.8 °C over the whole breeding range. Precipitation ranges from 0 to 188 mm with a mean of 53 mm. In the non-breeding season, the temperature ranges from 4.3 °C to 27 °C with a mean of 17 °C, and precipitation from 0 to 428 mm with a mean of 58 mm. Modelling the realized climatic niche of *C. pusilla* as a whole species for both seasons separately showed that the niche breadth was wider during the non-breeding season than during the breeding season (Fig. 2). The niche overlap between the seasons had a Schoener’s D value of 0.356, which according to the categories of Rödder & Engler (2011) constitutes low overlap.

**Figure 3:**
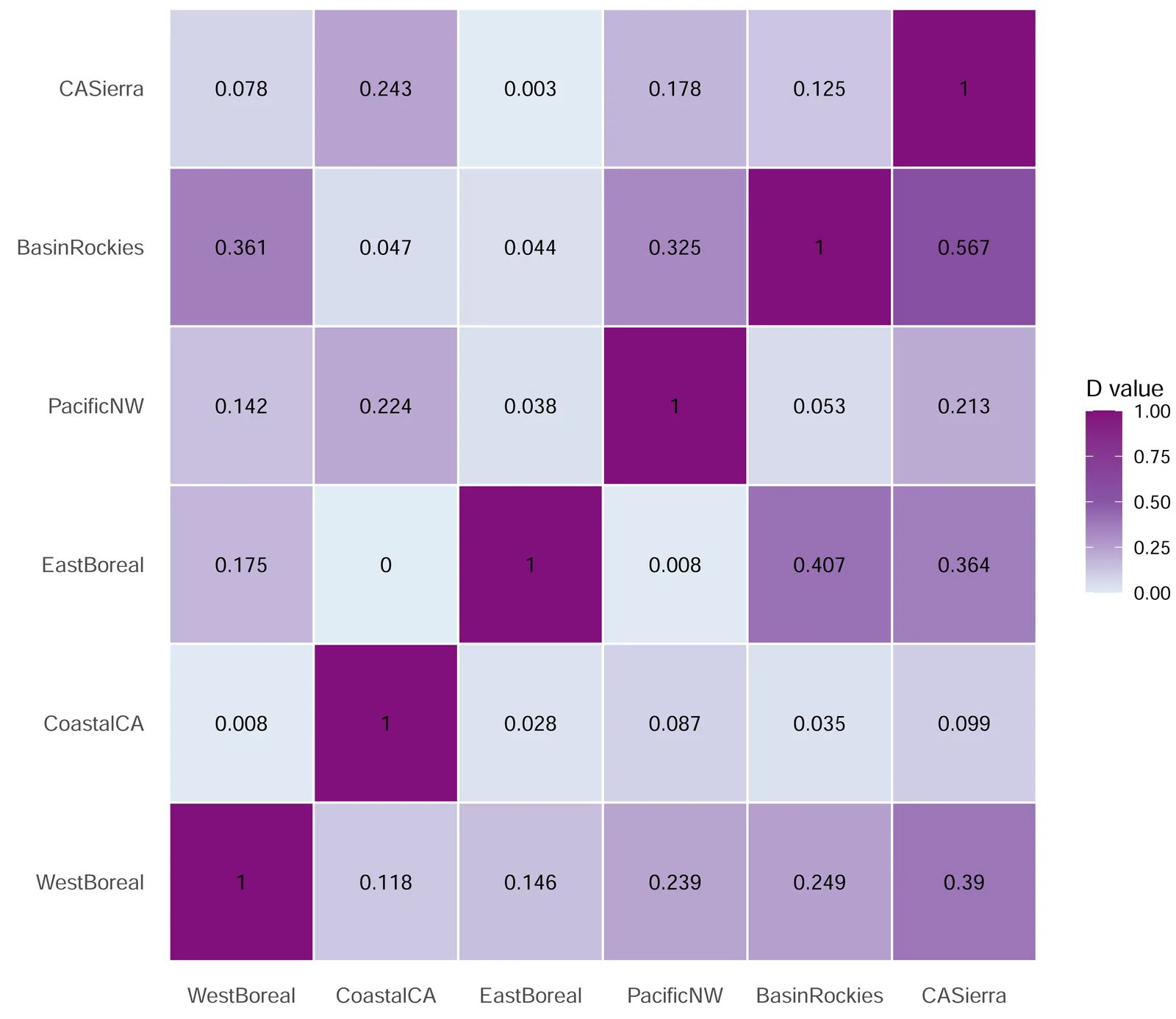
Realized climatic niche. Comparisons of upper part of the diagonal are the breeding realized climatic niche during the breeding season (above the diagonal) and non-breeding season (below the diagonal), as comparisons, while the lower part are the non-breeding realized climatic niche comparisons based on the overlap defined by the Shoener’s D similarity metric between all six genetically distinct populations of *Cardellina pusilla*.

At the population level, Western Boreal and Pacific Northwest had wider niche breadth for the breeding season than the non-breeding season, while for the rest of the populations the non-breeding season had wider niche breadth. The total niche breadth (breeding and non-breeding seasons combined) was widest in Western Boreal and narrowest in Pacific Northwest (Fig. 2). In general, there was low overlap of climatic niches between populations during the breeding season (*D* = 0–0.361; Fig. 3). The strongest overlap was between Basin Rockies and Western Boreal (0.361), followed by Basin Rockies and Pacific Northwest (0.325) and Coastal California and California Sierra (0.243). There was no overlap between Eastern Boreal and Coastal California and very low overlap between Western Boreal and Coastal California (0.008). In general, Eastern Boreal had the least similar climate preferences to the rest of the populations, with very low overlap (between 0 and 0.175) with the rest of the populations (Fig. 3). Coastal California’s breeding niche had stronger overlap with the populations that it is geographically closest to, though it had low overlap with Pacific Northwest (0.224) and California Sierra (0.243). There was a similar trend for California Sierra, with an overlap of 0.178 with Pacific Northwest, and 0.125 with Basin Rockies, showing that the western populations had some degree of overlap, albeit low, while Eastern Boreal was more differentiated from them (Fig. 3).

The realized climatic niches during the non-breeding season had stronger overlap among populations than breeding season niches (Fig. 3). Overall, there was low to moderate overlap between non-breeding climatic niches, but the range was broad (0.008 to 0.567; Fig. 3). The lowest overlap was between Eastern Boreal and Pacific Northwest (0.008). The non-breeding niche of Eastern Boreal had low overall overlap with the other groups (0.008−0.146), except with California Sierra (0.364) and Basin Rockies (0.407). California Sierra had the most similar non-breeding niche to the other populations, with overlap values ranging from 0.099 with Coastal California to 0.567 with Basin Rockies, which was the highest niche overlap between populations in the non-breeding season (Fig. 3).

**Figure 4:**
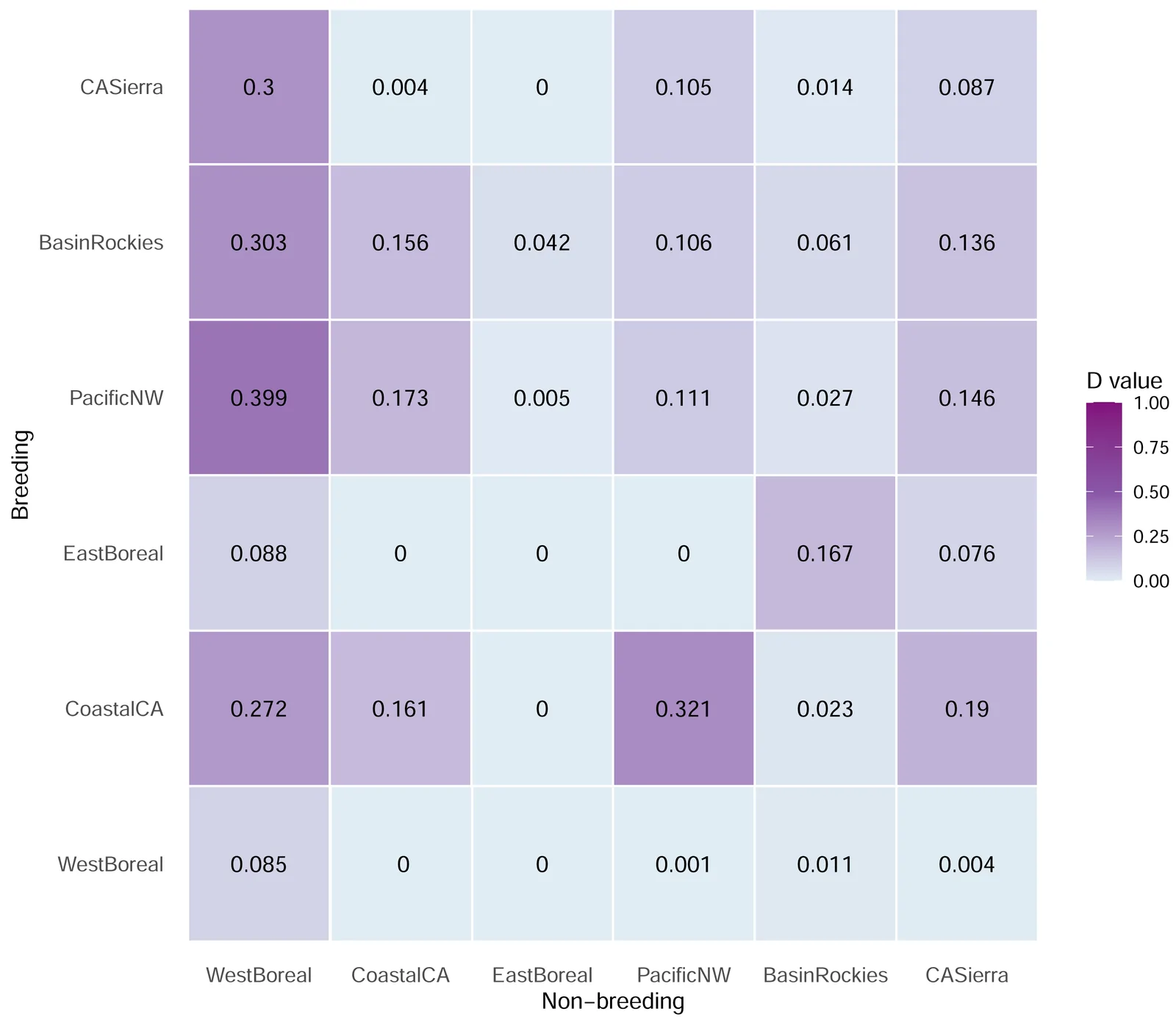
Breeding climatic niche *vs* non-breeding climatic niche comparisons based on the overlap defined by the Shoener’s D metric. Similarity of the breeding climatic conditions of each of the six populations if they switched their non-breeding final destination for that of another population.

Comparing the seasonal climatic preferences of each population (Fig. 4) showed low overlap between seasons for each population. The strongest niche overlap between seasons was for Coastal California, followed by Pacific Northwest (*D* = 0.161 and 0.111, respectively), and the lowest was that of Eastern Boreal (*D* = 0). The other comparisons from this analysis show that some populations could have a better climatic fit if they migrated to another population’s destination in the non-breeding grounds. For example, this was the case of Coastal California if it migrated to the Pacific Northwest non-breeding grounds (*D* = 0.321, compared with the observed seasonal niche overlap with its own population of 0.161). Other populations like Western Boreal would do worse migrating to any other population’s non-breeding grounds, while Basin Rockies could do better by using any other population’s non-breeding grounds except for Eastern Boreal (Fig. 4). Based on the niche similarity test (Supplemental Information 1), none of the seasonal comparisons of the climatic niche of the six populations were significant, meaning that they do not track or switch their niche better than random.

Hellinger’s I (Supplemental Information 2) showed similar patterns to D but with higher values. Basin Rockies and Pacific Northwest (*I* = 0.535) were the two populations with the strongest climatic niche overlap, followed by Western Boreal and Basin Rockies (*I* = 0.494). Eastern Boreal was still the most differentiated population in terms of climate but had low overlap with Western Boreal (*I* = 0.298). In the non-breeding season, as with D, there was high overlap between Basin Rockies and Western Boreal with California Sierra (*I* = 0.716 and 0.534, respectively). Eastern Boreal had higher overlap values with the rest of the populations except with Pacific Northwest (*I* = 0.008) and Coastal California (*I* = 0.114), which were the most strongly differentiated populations in the non-breeding season. We analyzed and discussed our data based on the D value because it is more stable and statistically robust (Broennimann et al., 2012).

In general, the position within the climatic space in both seasons showed that most of the populations preferred places with low precipitation (Fig. 2). A preference for higher precipitation areas by the Eastern Boreal population accounted for its lower overlap with the remaining populations. For Coastal California and California Sierra, whose breeding grounds had very low precipitation (means of 2 and 13 mm, respectively) there was an interesting shift in the non-breeding grounds where they experienced more precipitation (37–38 mm), resulting in a broader overall climatic niche. Temperature did not vary strongly between seasons for most populations, with the exception of Eastern Boreal, whose non-breeding grounds are hotter than its breeding grounds.

## Discussion

*Cardellina pusilla* makes a long-distance journey latitudinally twice a year, during which it experiences a wide range of climatic conditions in a variety of ecosystems (Ammon & Gilbert, 2020). We have shown here that there is variability within the seasonal climatic niches of *C. pusilla,* supporting the findings of Ruiz-Sánchez et al. (2015). For the species as a whole, the realized non-breeding niche is broader than the breeding niche, with a low overlap between them (Schoener’s *D* = 0.356). This might result in an effective climate switching ability, leading to the selection of different combinations of environmental conditions throughout the annual cycle (Alerstam & Bäckman, 2018; Gómez et al., 2016; Helbig, 2003; Joseph & Stockwell, 2000; Nakazawa et al., 2004). A wider non-breeding niche and the ability to exploit different climates in each season could be related to the generalist habits in the non-breeding season, which has been observed in other migratory birds (Hutto, 1995). Meanwhile, comparing the realized climatic niches among genetically distinct populations revealed variation that would have otherwise been overlooked (Mota-Vargas & Rojas-Soto, 2016; Smith et al., 2019). We identified clear differentiation of the breeding niche of the Eastern Boreal population from the rest of the populations (Fig. 3). Despite non-breeding niches being more similar among populations than breeding niches, the non-breeding niche of the Eastern Boreal population still had low overlap with most of the other populations (D between 0.008−0.407). The observed ecological distinctiveness of the Eastern Boreal population may help shed light on the genetic distinctiveness of this group shown by previous work (*F*_*ST*_ = 0.41−0.68; Ruegg et al., 2014). When analyzing differences in the realized climatic niches across seasons, we found that some populations could actually do better migrating to the non-breeding areas of another population; such is the case of Coastal California migrating to the Pacific Northwest non-breeding destination and Pacific Northwest migrating to the Western Boreal destination (Fig. 4).

The genetic variation within *Cardellina pusilla* has been studied for many years now (Clegg et al., 2003; Irwin, Irwin & Smith, 2011; Kimura, Clegg & Lovette, 2002; Ruegg et al., 2014), but the potential of ecological heterogeneity as an influential factor for the differentiation of the six genetically distinct populations has not been well understood. We found ecological differentiation of the Eastern Boreal population, which has narrow niche breadth both on the breeding and the non-breeding grounds that might be the result of a distribution delimited to habitats with a combination of higher average precipitation levels and higher temperatures that correspond to the extensive, shrub-dominated boreal wetlands and riparian thickets of eastern Canada (Ammon & Gilbert, 2020). This habitat matrix, dominated by dense understories of alder (*Alnus*) and willow (*Salix*), fundamentally differs from the breeding habitats of western populations (Ammon & Gilbert, 2020; Ruegg et al., 2014; Ruiz-Sánchez et al., 2015). This aligns with what Ruiz-Sánchez et al. (2015) showed, that partial differentiation of their ecological niches is related with the narrow size of the niche of the eastern group, thus supporting the hypothesis that Eastern Boreal is a cryptic species within the *C. pusilla* complex and that its taxonomic status should be reevaluated (Irwin, Irwin & Smith, 2011; Ruegg et al., 2014; Ruiz-Sánchez et al., 2015). Future research into the potential cryptic species status of the Eastern Boreal population should focus on the potential for gene flow/assortative mating between eastern and western populations (Irwin, Irwin & Smith, 2011; Toews, 2017; Wang & Bradburd, 2014).

In addition to differences between the Eastern Boreal and western populations, we also showed climate differentiation on the breeding areas of the Coastal California population from the rest of the populations, especially with Western Boreal, Eastern Boreal and Basin Rockies. This is noteworthy due to the very different ecosystems they use on the breeding grounds, particularly in western North America (Ruegg et al., 2017), breeding in fog-drenched coastal scrub and early-successional forests where they exhibit a singular nesting behavior, constructing nests up to 1.5 m above ground (an exception to the ground-nesting behavior observed in the rest of the species), a possible adaptation to local predation pressures or microclimate (Ammon & Gilbert, 2020). Coastal California had low overlap with California Sierra and Pacific Northwest on the breeding grounds. This is consistent with the subspecies *C. pusilla chryseola*, and Ruiz-Sánchez et al. (2015) overlooked this distinction by clustering all western populations together as distinct from an eastern population. The differentiation of their breeding climatic niche is important to understand the splitting of each population and the separation of their distribution ranges (Fandos & Tellería, 2020; Laube, Graham & Böhning-Gaese, 2015). Further sampling would add more information on these three populations, which are not very well differentiated genetically (Irwin, Irwin & Smith, 2011; Ruegg et al., 2014). Despite the fact that as a migratory bird they have great dispersal ability, each population appears to have strong philopatry and to be selecting areas in the breeding grounds that have specific climate features (Fig. 3). These features act as a mosaic of heterogeneous environmental conditions, which could be one of various factors leading to the differentiation of populations (Bay et al., 2018; Kawecki & Ebert, 2004; Kozak & Wiens, 2007; Wang & Bradburd, 2014; Winger et al., 2019).

The low seasonal climatic niche overlap observed in the species *C. pusilla* as a whole is consistent across the genetic groups: most populations had very low overlap between breeding and non-breeding niches (Fig. 4). But there are some ecological and evolutionary features of the natural history of the populations that may be obscured considering the species level alone (Mota-Vargas & Rojas-Soto, 2016; Smith et al., 2019). Tracking the climatic niche through seasons as a migratory strategy can promote the evolution of narrow and specialized niches when natural selection favors the same traits on both the breeding and the non-breeding grounds, whereas the ability to switch the niche may result in a generalist niche overall if natural selection acts in opposite directions at each stage of the annual cycle (Robinson et al., 2009; Webster & Marra, 2004). None of the populations behave as niche trackers, and they tend to act more like niche switchers due to the low seasonal climatic niche overlap of the six populations. Even so, the observed D value reveals that some populations would achieve a better climatic fit (observed D value) if they migrated to another population’s non-breeding grounds (Fig. 4). For example, Coastal California has a very low seasonal climatic niche overlap (*D* = 0.161), but migrating to the non-breeding grounds of the Pacific Northwest (*D* = 0.321) they would encounter climatic conditions that are more similar to those on their own breeding grounds. This would also happen if Pacific Northwest (*D* = 0.111) migrated to Western Boreal’s non-breeding grounds (*D* = 0.399). This suggests that on a continental scale, energy efficiency (optimizing the balance between accessing available resources and movement cost) is likely driving the migration route (Somveille et al., 2024). In other words, although a population could reach a non-breeding area with a more similar climate to their breeding grounds, the migratory route is optimized for travel and resource access efficiency rather than maximizing climate similarity (Somveille et al., 2024). In addition to this, there is evidence that the non-breeding distribution of the six populations has been stable since the mid-20th century despite environmental changes, suggesting high non-breeding site fidelity that is probably learned or genetically coded (Turbek et al., 2025). This niche conservatism could also be acting against the optimal climatic fit to experience similar climates through seasons. By extension, if the climate in the non-breeding areas changes (*e.g*. drought), a population would likely not be readily able to switch to an alternative non-breeding destination with better climate. Thus, climate change vulnerability is population-specific and is determined by migratory fidelity (Saracco & Rubenstein, 2020; Winger et al., 2019).

Taken as a whole, this generates an interesting collection of results. Each population of *C. pusilla* has different preferences, which also vary between seasons. The fact that Eastern Boreal has narrow, non-overlapping niches suggests that even though they select specific conditions and are dependent on boreal wetlands during the breeding season, they have the plasticity to switch to very different and specialized conditions when they establish in the pine-oak forests and cloud forests in the non-breeding grounds, supporting the idea that some biotic and abiotic variables become more relevant in one life stage or the other due to phenological changes (Ammon & Gilbert, 2020; Bolnick et al., 2003; Ponti & Sannolo, 2022; Rushing et al., 2020; Sexton et al., 2017). More genetic samples from the non-breeding grounds would help to corroborate these results. The low overlap of climatic features in the breeding grounds and the higher overall overlap in the non-breeding grounds among populations could be an indication that the genetically distinct populations are adapting locally to the environmental heterogeneity in the breeding areas, but that does not reduce their capacity to exploit different climatic resources in the non-breeding areas (Gilroy et al., 2016; Ponti et al., 2020; Somveille, Manica & Rodrigues, 2019; Winger et al., 2019). Other studies have shown that the capacity to exploit different climatic features through the annual cycle of a species might be less related to climatic conditions and more to the habitat selection and in other cases to energy efficiency (Dufour et al., 2020; Eyres et al., 2020; Ponti et al., 2020; Somveille et al., 2024; Zurell et al., 2018). This could be indicative of the plasticity of each population and the appearance of migratory behavior in the species as an adaptation to extreme seasonality in which breeding-site fidelity is an advantage for survival, despite the climatic characteristics they encounter on the non-breeding grounds (Gilroy et al., 2016; Winger et al., 2019). The fact that migratory behavior reappeared in the evolutionary history of the clade from a nonmigratory ancestor and is immersed in a large clade of sedentary (Neotropical resident) species (Lovette et al., 2010; Winger, Lovette & Winkler, 2012; Winger, Barker & Ree, 2014), might suggest that the divergence of the populations could be related to the specialization of their climatic preferences on the breeding grounds (La Sorte, Fink & Johnston, 2018; Winger et al., 2019). In turn, the fidelity of each population to its non-breeding sites might be important to increase survival and fitness, which could be selected across generations and support the selection of those areas and their climatic features (Winger et al., 2019)

The varying migratory patterns and degrees of specialization across the annual cycle are important information for the management of populations amidst rapid global change and should be population-specific, since their climate change vulnerability is determined by their migratory fidelity (Marra et al., 2015; Runge et al., 2014; Saracco & Rubenstein, 2020). Conservation efforts should focus on the protection of unique breeding habitats like that of Eastern Boreal, a highly differentiated population with a unique climatic niche that makes it very sensitive to drastic changes in the breeding and non-breeding climates (Bay et al., 2018; La Sorte, Fink & Johnston, 2018; Runge et al., 2014). Coastal California has a similar situation, especially in the non-breeding grounds, as it has a very differentiated climatic niche. Although it potentially could migrate to other areas, energy efficiency and site fidelity is what drives their migration (Saracco & Rubenstein, 2020). Furthermore, Coastal California, Pacific Northwest and California Sierra (subspecies *C. pusilla chryseola*) share a vulnerable non-breeding area in Baja California Sur and Northwestern Mexico that is recognized as a genetically important non-breeding ground (Ruegg et al., 2020). For Coastal California especially, these are the only places they spend the non-breeding season, unlike California Sierra and Pacific Northwest which migrate further south throughout western Mexico and Central America. Thus, special attention must be paid to conserving the non-breeding areas of these populations because it has been shown that their survival is negatively related to drought in the non-breeding season, a phenomenon that is becoming more common every year (Saracco & Rubenstein, 2020). Lastly, Basin Rockies has a small and declining population size, making it a priority for further conservation (Gilroy et al., 2016; Rosenberg et al., 2019; Ruegg et al., 2020; Turbek et al., 2025). Conservation efforts must also be made in the breeding areas in the face of the recent exacerbated wildfires (Kittelberger, Miller & Şekercioğlu, 2022).

We found here that studying the realized climatic niche at the infraspecific level is a helpful tool to understand how populations respond in a heterogeneous environment throughout their annual cycle and if the response can inform on processes of genetic differentiation. Our analyses reveal that climatic niche overlap in *Cardellina pusilla*’s genetically distinct populations not only describes ecological similarities, but also gives relevant information on how population structure has been shaped by the interaction between philopatry, stability of non-breeding grounds over time, and differential selection of climate conditions in the breeding grounds. The extent to which the population-specific differences in niche use noted herein may lead to further differentiation within the species is an important area for future investigation. In addition to this, management and conservation strategies should focus on protecting historically used non-breeding areas and consider the populations’ limited capacity to readjust their migratory destinations in the face of climate change. Thus, conserving the migratory corridors by reducing anthropogenic threats in both breeding and non-breeding areas are key steps to ensure the survival of genetically distinct populations.

##  Supplemental Information

10.7717/peerj.21540/supp-1 Supplemental Information 1 Supplemental information on niche similarity analysis data and Hellinger’s I values

## Additional Information and Declarations

### Field Study Permissions

The following information was supplied relating to field study approvals (*i.e.*, approving body and any reference numbers): The field collection permit was provided by Instituto Nacional de Ecologia, SEMARNAT, Mexico (FAUT-0169).

### Data Availability

The following information was supplied regarding data availability: Genomic data from the 107 new genotype samples is available at Dryad: Rodríguez Otero, Genaro; Genaro Rodríguez; Bossu, Christen; Somveille, Marius et al. (2026). Climatic niche variation in genetically distinct populations throughout the annual cycle for a migratory parulid bird, Cardellina pusilla [Dataset]. Dryad. https://doi.org/10.5061/dryad.4j0zpc8qx.

## References

[ref-1] Alerstam T, Bäckman J (2018). Ecology of animal migration. Current Biology.

[ref-2] Alvarado AH, Bossu CM, Harrigan RJ, Bay RA, Nelson ARP, Smith TB, Ruegg KC (2022). Genotype–environment associations across spatial scales reveal the importance of putative adaptive genetic variation in divergence. Evolutionary Applications.

[ref-3] Ammon EM, Gilbert WM, Rodewald PG (2020). Wilson’s Warbler (*Cardellina pusilla*), version 1.0. Birds of the World.

[ref-4] Araújo MB, Thuiller W, Yoccoz N (2009). Reopening the climate envelope reveals macroscale associations with climate in European birds. Proceedings of the National Academy of Sciences of the United States of America.

[ref-5] Bay R, Harrigan RJ, Underwood VL, Gibbs HL, Smith TB, Ruegg K (2018). Genomic signals of selection predict climate-driven population declines in a migratory bird. Science.

[ref-6] Bivand R, Keitt T, Rowlingson B (2022). https://cran.r-project.org/src/contrib/Archive/rgdal/.

[ref-7] Bivand RS, Pebesma E, Gomez-Rubio V (2013). Applied spatial data analysis with R.

[ref-8] Bolnick DI, Svanbäck R, Fordyce JA, Yang LH, Davis JM, Hulsey CD, Forister ML (2003). The ecology of individuals: incidence and implications of individual specialization. The American Naturalist.

[ref-9] Broennimann O, Fitzpatrick MC, Pearman PB, Petitpierre B, Pellissier L, Yoccoz NG, Thuiller W, Fortin MJ, Randin C, Zimmermann NE, Graham CH, Guisan A (2012). Measuring ecological niche overlap from occurrence and spatial environmental data. Global Ecology and Biogeography.

[ref-10] Chen Y, Jiang Z, Fan P, Ericson PGP, Song G, Luo X, Lei F, Qu Y (2022). The combination of genomic offset and niche modelling provides insights into climate change-driven vulnerability. Nature Communications.

[ref-11] Clegg SM, Kelly JF, Kimura M, Smith TB (2003). Combining genetic markers and stable isotopes to reveal population connectivity and migration patterns in a Neotropical migrant, Wilson’s warbler (*Wilsonia pusilla*). Molecular Ecology.

[ref-12] De Saix MG, Bullock LP, Eckert AJ, Viverette CB, Boves TJ, Tonra JA, Dyer RJ (2019). Population assignment reveals low migratory connectivity in a weakly structured songbird. Molecular Ecology.

[ref-13] Di Cola V, Broennimann O, Petitpierre B, Breiner FT, D’Amen M, Randin C, Engler R, Pottier J, Pio D, Dubuis A, Pellissier L, Mateo RG, Hordijk W, Salamin N, Guisan A (2017). ecospat: an R package to support spatial analyses and modeling of species niches and distributions. Ecography.

[ref-14] Dufour P, Descamps S, Chantepie S, Renaud J, Guéguen M, Schiffers K, Thuiller W, Lavergne S (2020). Reconstructing the geographic and climatic origins of long-distance bird migrations. Journal of Biogeography.

[ref-15] Edelaar P, Bolnick DI (2012). Non-random gene flow: an underappreciated force in evolution and ecology. Trends in Ecology and Evolution.

[ref-16] Eyres A, Böhning-Gaese K, Fritz SA (2017). Quantification of climatic niches in birds: adding the temporal dimension. Journal of Avian Biology.

[ref-17] Eyres A, Böhning-Gaese K, Orme CDL, Rahbek C, Fritz SA (2020). A tale of two seasons: the link between seasonal migration and climatic niches in passerine birds. Ecology and Evolution.

[ref-18] Fandos G, Tellería JL (2020). Seasonal niche-tracking behaviour of two partially migratory passerines. Ibis.

[ref-19] Fick SE, Hijmans RJ (2017). WorldClim 2: new 1km spatial resolution climate surfaces for global land areas. International Journal of Climatology.

[ref-20] Fink D, Auer T, Johnston A, Strimas-Mackey M, Ligocki S, Robinson O, Hochachka W, Jaromczyk L, Rodewald A, Wood C, Davies I, Spencer A (2022). eBird Status and Trends, Data Version: 2021; Released: 2022. Cornell Lab of Ornithology.

[ref-21] Fitzpatrick MC, Keller SR (2015). Ecological genomics meets community-level modelling of biodiversity: mapping the genomic landscape of current and future environmental adaptation. Ecology Letters.

[ref-22] GBIF.org (2022). GBIF Occurrence Download.

[ref-23] Gilroy JJ, Gill JA, Butchart SH, Jones VR, Franco AM (2016). Migratory diversity predicts population declines in birds. Ecology Letters.

[ref-24] Gómez C, Tenorio EA, Montoya P, Cadena CD (2016). Niche-tracking migrants and niche-switching residents: evolution of climatic niches in the New World warblers (Parulidae). Proceedings of the Royal Society B: Biological Sciences.

[ref-25] Greenberg RS, Lovette IJ, Fitzpatrick JW (2016). Bird communities. The Cornell Lab of Ornithology’s Handbook of Bird Biology.

[ref-26] Gutiérrez Illán J, Wang G, King DT, Cunningham FL (2022). Seasonal variation and tracking of climate niche of a migratory bird. Global Ecology and Conservation.

[ref-27] Helbig AJ, Berthold P, Gwinner E, Sonnenschein E (2003). Evolution of bird migration: a phylogenetic and biogeographic perspective. Avian migration.

[ref-28] Hijmans RJ (2020). https://CRAN.R-project.org/package=raster.

[ref-29] Hijmans RJ (2022). https://CRAN.R-project.org/package=terra.

[ref-30] Hutto R, Wilson MH, Sader SA (1995). Can patterns of vegetation change in western Mexico explain population trends in western neotropical migrants?. Conservation of neotropical migratory birds in Mexico. Misc. Publ. 727.

[ref-31] Irwin DE, Irwin JH, Smith TB (2011). Genetic variation and seasonal migratory connectivity in Wilson’s warblers (*Wilsonia pusilla*): species-level differences in nuclear DNA between western and eastern populations. Molecular Ecology.

[ref-32] Janzen DH (1967). Why mountain passes are higher in the tropics?. The American Naturalist.

[ref-33] Joseph L, Stockwell D (2000). Temperature-based models of the migration of Swainson’s flycatcher (*Myiarchus swainsonii*) across South America: a new use for museum specimens of migratory birds. Proceedings of the Academy of Natural Sciences of Philadelphia.

[ref-34] Kawecki TJ, Ebert D (2004). Conceptual issues in local adaptation. Ecology Letters.

[ref-35] Kimura M, Clegg SM, Lovette IJ, Holder KR, Girman DJ, Milá B, Wade P, Smith TB (2002). Phylogeographical approaches to assessing demographic connectivity between breeding and overwintering regions in a Nearctic-Neotropical warbler (*Wilsonia pusilla)*. Molecular Ecology.

[ref-36] Kirby JS, Stattersfield AJ, Butchart SHM, Evans MI, Grimmett RFA, Jones VR, O’Sullivan J, Tucker GM, Newton I (2008). Key conservation issues for migratory land- and waterbird species on the world’s major flyways. Bird Conservation International.

[ref-37] Kittelberger KD, Miller MK, Şekercioğlu ÇH (2022). Fall bird migration in western North America during a period of heightened wildfire activity. Avian Conservation and Ecology.

[ref-38] Kozak KH, Wiens JJ (2007). Climatic zonation drives latitudinal variation in speciation mechanisms. Proceedings of the Royal Society B: Biological Sciences.

[ref-39] La Sorte FA, Fink D, Johnston A (2018). Seasonal associations with novel climates for North American migratory bird populations. Ecology Letters.

[ref-40] Laube I, Graham CH, Böhning-Gaese K (2015). Niche availability in space and time: migration in *Sylvia* warblers. Journal of Biogeography.

[ref-41] Lovette IJ, Pérez-Emán JL, Sullivan JP, Banks RC, Fiorentino I, Córdoba-Córdoba S, Echeverry-Galvis M, Barker FK, Burns KJ, Klicka J, Lanyon SM, Bermingham E (2010). A comprehensive multilocus phylogeny for the wood-warblers and a revised classification of the Parulidae (Aves). Molecular Phylogenetics and Evolution.

[ref-42] Marra P, Cohen EB, Loss SR, Rutter JE, Tonra CM (2015). A call for full annual cycle research in animal ecology. Biology Letters.

[ref-43] Martínez-Meyer E, Peterson AT, Navarro-Sigüenza AG (2004). Evolution of seasonal ecological niches in the *Passerina* buntings (Aves: Cardinalidae). Proceedings of the Royal Society B: Biological Sciences.

[ref-44] Moran BM, Anderson EC (2018). Bayesian inference from the conditional genetic stock identification model. Canadian Journal of Fisheries and Aquatic Sciences.

[ref-45] Mota-Vargas C, Rojas-Soto OR (2016). Taxonomy and ecological niche modeling: implications for the conservation of Wood patridges (genus *Dendrortyx*). Journal for Nature Conservation.

[ref-46] Nakazawa Y (2013). Niche breadth, environmental landscape, and physical barriers: their importance as determinants of species distributions. Biological Journal of the Linnean Society.

[ref-47] Nakazawa Y, Peterson AT, Martínez-Meyer E, Navarro-Sigüenza AG (2004). Seasonal niches of migratory birds: implications for the evolution of migration. The Auk.

[ref-48] Nosil P, Vines TH, Funk DJ (2005). Perspective: reproductive isolation caused by natural selection against immigrants from divergent habitats. Evolution.

[ref-49] Ochoa-González A, Rojas-Soto OR, Prieto-Torres DA, Arizmendi MC, Navarro-Sigüenza AG (2023). At home in the tropics: seasonal niche-tracking by the Yellow-green Vireo, Vireo flavoviridis, an intratropical migrant. Revista Mexicana de Biodiversidad.

[ref-50] Pegan TM, Kimmitt AA, Benz BW, Weeks BC, Aubry Y, Burg TM, Hudon J, Jones AW, Kirchman JJ, Ruegg KC, Winger BM (2025). Long-distance seasonal migration to the tropics promotes genetic diversity but not gene flow in boreal birds. Nature Ecology & Evolution.

[ref-51] Ponti R, Arcones A, Ferrer X, Vieites DR (2020). Seasonal climatic niches diverge in migratory birds. Ibis.

[ref-52] Ponti R, Sannolo M (2022). The importance of including phenology when modelling species ecological niche. Ecography.

[ref-53] R Core Team (2023). https://www.R-project.org/.

[ref-54] Robinson RA, Crick HQP, Learmonth JA, Maclean IMD, Thomas CD, Bairlein F, Forchhammer MC, Francis CM, Gill JA, Godley BJ, Harwood J, Hays GC, Huntley B, Hutson AM, Pierce GJ, Rehfisch MM, Sims DW, Santos MB, Sparks TH, Stroud DA, Visser ME (2009). Travelling through a warming world: climate change and migratory species. Endangered Species Research.

[ref-55] Rödder D, Engler JO (2011). Quantitative metrics of overlaps in Grinnellian niches: advances and possible drawbacks. Global Ecology and Biogeography.

[ref-56] Rosenberg KV, Dokter AM, Blancher PJ, Sauer JR, Smith AC, Smith PA, Stanton JC, Panjabi A, Helft L, Parr M (2019). Decline of the North American avifauna. Science.

[ref-57] Rueda-Hernández R, Bossu CM, Smith TB, Contina A, Canales Del Castillo R, Ruegg K, Hernández-Baños BE (2023). Winter connectivity and leapfrog migration in a migratory passerine. Ecology and Evolution.

[ref-58] Ruegg KC, Anderson EC, Harrigan RJ, Paxton KL, Kelly JF, Moore F, Smith TB (2017). Genetic assignment with isotopes and habitat suitability (GAIAH), a migratory bird case study. Methods in Ecology and Evolution.

[ref-59] Ruegg K, Anderson EC, Paxton KL, Apkenas V, Lao S, Siegel RB, De Sante DF, Moore F, Smith TB (2014). Mapping migration in a songbird using high-resolution genetic markers. Molecular Ecology.

[ref-60] Ruegg K, Anderson EC, Somveille M, Bay RA, Whitfield M, Paxton EH, Smith TB (2021). Linking climate niches across seasons to assess population vulnerability in a migratory bird. Global Change Biology.

[ref-61] Ruegg K, Harrigan R, Saracco J, Smith TB, Taylor CM (2020). A genoscape-network model for conservation prioritization in a migratory bird. Conservation Biology.

[ref-62] Ruiz-Sánchez A, Renton K, Landgrave-Ramírez R, Mora-Aguilar EF, Rojas-Soto O (2015). Ecological niche variation in the Wilson’s warbler *Cardellina pusilla* complex. Journal of Avian Biology.

[ref-63] Runge CA, Martin TG, Possingham HP, Willis SG, Fuller RA (2014). Conserving mobile species. Frontiers in Ecology and the Environment.

[ref-64] Rushing CS, Royle JA, Ziolkowski DJ, Pardieck KL (2020). Migratory behavior and winter geography drive differential range shifts of eastern birds in response to recent climate change. Proceedings of the National Academy of Sciences of the United States of America.

[ref-65] Saracco JF, Rubenstein M (2020). Integrating broad-scale data to assess demographic and climatic contributions to population change in a declining songbird. Ecology and Evolution.

[ref-66] Savolainen O, Lascoux M, Merilä J (2013). Ecological genomics of local adaptation. Nature Reviews Genetics.

[ref-67] Seutin G, White BN, Boag PT (1991). Preservation of avian blood and tissue samples for DNA analyses. Canadian Journal of Zoology.

[ref-68] Sexton JP, Montiel J, Shay JE, Stephens MR, Slatyer RA (2017). Evolution of ecological niche breadth. Annual Review of Ecology, Evolution, and Systematics.

[ref-69] Smith AB, Godsoe W, Rodríguez-Sánchez F, Wang HH, Warren D (2019). Niche estimation above and below the species level. Trends in Ecology and Evolution.

[ref-70] Somveille M, Bossu CM, De Saix MG, Alvarado AH, Gómez Villaverde S, Rodríguez Otero G, Hernández-Baños BE, Ruegg K (2024). Broad-scale seasonal climate tracking is a consequence, not a driver, of avian migratory connectivity. Ecology Letters.

[ref-71] Somveille M, Manica A, Rodrigues ASL (2019). Where the wild birds go: explaining the differences in migratory destinations across terrestrial bird species. Ecography.

[ref-72] Somveille M, Rodrigues ASL, Manica A (2018). Energy efficiency drives the global seasonal distribution of birds. Nature Ecology & Evolution.

[ref-73] Toews DPL (2017). Habitat suitability and the constraints of migration in New World warblers. Journal of Avian Biology.

[ref-74] Turbek SP, Polich A, Bossu CM, Rayne C, Carpenter A, Rodríguez Otero G, Gómez Villaverde S, Rodríguez Vásquez F, Hernández-Baños BE, McCormack J, Ruegg K (2025). Genetic analysis of museum samples suggests temporal stability in the Mexican nonbreeding distribution of a neotropical migrant. Journal of Avian Biology.

[ref-75] Turbek SP, Scordato ESC, Safran RJ (2018). The role of seasonal migration in population divergence and reproductive isolation. Trends in Ecology & Evolution.

[ref-76] Venables WN, Ripley BD (2002). Modern applied statistics with S.

[ref-77] Wang IJ (2013). Examining the full effects of landscape heterogeneity on spatial genetic variation: a multiple matrix regression approach for quantifying geographic and ecological isolation. Evolution.

[ref-78] Wang IJ, Bradburd GS (2014). Isolation by environment. Molecular Ecology.

[ref-79] Warren DL, Glor RE, Turelli M (2008). Environmental niche equivalency *versus* conservatism: quantitative approaches to niche evolution. Evolution.

[ref-80] Webster MS, Marra PP (2004). Birds of two worlds—The ecology and evolution of migration.

[ref-81] Webster M, Marra P, Haig S, Bensch S, Holmes R (2002). Links between worlds: unravelling migratory connectivity. Trends in Ecology & Evolution.

[ref-82] Wiens JJ (2004). Speciation and ecology revisited: phylogenetic niche conservatism and the origin of the species. Evolution.

[ref-83] Winger BM, Auteri GG, Pegan TM, Weeks BC (2019). A long winter for the Red Queen: rethinking the evolution of seasonal migration. Biological Reviews.

[ref-84] Winger BM, Barker FK, Ree RH (2014). Temperate origins of long-distance seasonal migration in New World songbirds. Proceedings of the National Academy of Sciences of the United States of America.

[ref-85] Winger BM, Lovette IJ, Winkler DW (2012). Ancestry and evolution of seasonal migration in the Parulidae. Proceedings of the Royal Society B: Biological Sciences.

[ref-86] Zurell D, Gallien L, Graham CH, Zimmermann NE (2018). Do long-distance migratory birds track their niche through seasons?. Journal of Biogeography.

