# Supplementary material for: Climatic niche variation in genetically distinct populations throughout the annual cycle for a migratory parulid bird, Cardellina pusilla
Source: PeerJ. 2026 Aug 17;14:e21540. doi: 10.7717/peerj.21540 (PMC13492351; doi:10.7717/peerj.21540)
Supplement: Supplementary file 1 [file peerj-14-21540-s001.docx]

**SUPPLEMENTAL INFORMATION**

Supplemental information 1

S. I. 1 Niche similarity tests for each population through the annual cycle. p values are non significant for every population

Supplemental information 2

a)

|  | West  Boreal | Coastal  CA | EastBoreal | PacificNW | Basin  Rockies | CA  Sierra |
| --- | --- | --- | --- | --- | --- | --- |
| WestBoreal | 0.156 | -2.22E-16 | -2.22E-16 | 0.001 | 0.018 | 0.011 |
| CoastalCA | 0.518 | 0.203 | 2.22E-16 | 0.478 | 0.109 | 0.372 |
| EastBoreal | 0.137 | 2.22E-16 | -2.22E-16 | 2.22E-16 | 0.214 | 0.096 |
| PacificNW | 0.597 | 0.288 | 0.012 | 0.172 | 0.096 | 0.275 |
| BasinRockies | 0.427 | 0.324 | 0.079 | 0.164 | 0.134 | 0.219 |
| CASierra | 0.426 | 0.012 | 2.22E-16 | 0.184 | 0.029 | 0.127 |

b)

|  | West  Boreal | Coastal  CA | EastBoreal | PacificNW | Basin  Rockies | CASierra |
| --- | --- | --- | --- | --- | --- | --- |
| WestBoreal | 1 | 0.016 | 0.298 | 0.261 | 0.494 | 0.151 |
| CoastalCA |  | 1 | 2.22E-16 | 0.351 | 0.096 | 0.419 |
| EastBoreal |  |  | 1 | 0.059 | 0.084 | 0.004 |
| PacificNW |  |  |  | 1 | 0.535 | 0.306 |
| BasinRockies |  |  |  |  | 1 | 0.207 |
| CASierra |  |  |  |  |  | 1 |

c)

|  | West  Boreal | CoastalCA | EastBoreal | PacificNW | Basin  Rockies | CASierra |
| --- | --- | --- | --- | --- | --- | --- |
| WestBoreal | 1 | 0.310 | 0.263 | 0.449 | 0.423 | 0.533 |
| CoastalCA |  | 1 | 0.114 | 0.162 | 0.180 | 0.296 |
| EastBoreal |  |  | 1 | 0.007 | 0.504 | 0.412 |
| PacificNW |  |  |  | 1 | 0.166 | 0.392 |
| BasinRockies |  |  |  |  | 1 | 0.716 |
| CASierra |  |  |  |  |  | 1 |

S. I. 2 a) 6 x 6 matrix with each Hellinger’s I values of every population’s realized breeding niche (rows) and those for the non-breeding season (columns), to obtain the niche overlap for all possible shifts in destination. b) Hellinger’s I values for breeding climatic niche comparisons between every population. c) I values for non-breeding climatic niche comparisons between every population
